# Locomotor Ataxia; Its Recent Pathology and Treatment

**Published:** 1898-07

**Authors:** F. Guillemont

**Affiliations:** Niagara Falls, N. Y.


### LOCOMOTOR ATAXIA; ITS RECENT PATHOLOGY AND TREATMENT.

By F. GUILLEMONT, M. D., Niagara Falls, N. Y.

TO COMPARE what is at present known of the pathology of locomotor ataxia with the theories generally accepted up to within a few years, is one demonstration of the phenomenal advancement made in all branches of science within the past decade. Scientific research has many dark corners to lighten up in all its branches, but in none has it more scope than in that of neuropathy. Indeed, up to comparatively a few years ago, the whole nervous system, as regards its physiology and pathology, was almost a closed book and that much-abused but convenient theory of sclerosis was trimmed and molded so as to fit into the pathology of all chronic nervous diseases. We were taught to look upon tabes as a sclerosis affecting the columns of Burdach and Gall and, in the earlier stages, a recent writer described a neuritis of the posterior spinal roots.

The vivid picture set before us during our college course and still holding prominence in many of the more recent text-books, is one not easily forgotten; the inflammatory action within the nerve sheath with its exudation of lymph, round-celled infiltration of the neuroglia and proliferation of connective tissue elements, followed by organisation of cicatricial tissue with consequent shrinkage and choking of the delicate nerve structures; the continuance of these pathological changes producing destruction of function after function and finally the picture presents a case utterly helpless beyond the one false hope of the connective-tissue-absorbing properties of iodide of potash.

The later conceptions of the pathology of tabes are mostly due to Woldyer's demonstration of the neuron and its disassociation from the central nervous system. Contained within the posterior spinal ganglion we find a small body called the neuron or neuron-body; from this are given off nerve filaments extending in one direction along the posterior spinal roots and up the spinal cord, entering at Burdach's column, and in the lumbar cord, crossing over to Gall's column, up which it extends to the oblongata, when it terminates by numerous filaments called arborisation. This branch is called the spinal neuraxon. In the other direction these fibers are directed peripherally along the spinal nerves and terminate in a delicate arborisation at the muscle-spindle. This branch is called the peripheral neuraxon.

---

1. Read at the Niagara Falls Academy of Medicine, April, 1898.

Perhaps a few words on the muscle-spindle will help clear the way for further explanation. Although this body has been known for over half a century and its functions variously interpreted, only recently has it been demonstrated that it is really an organ of muscle sense. It has been described as an organ of sensation within an organ of motion and to it have been attributed the posture and weight senses. It is a spindle-shaped body, about one-third the size of the surrounding muscular fibers. It is contained within a sheath and is composed of lymph and a few delicate fibers within the sheath. Each spindle has an artery and a vein and also a nerve supply. The nerves are afferent in direction and constitute the peripheral branch of the neuron. Now, with a picture before us of the neuron-body, its spinal and peripheral branches and also the muscle-spindle, we may proceed to apply these later anatomical developments to an explanation of the changes that take place in locomotor ataxia.

Graphically speaking, we may consider the neuron, its branches and the muscle-spindle as one organ ; an organ that governs coördination by virtue of its muscular sense and this organ is independent of brain control, much the same as are the reflexes. Take, for instance, a condition of generally lowered nutrition of the nervous system, which may result either from predisposition—hereditary or acquired—or from excessive mental strain or, which is perhaps more frequently the case, presence of toxins in the blood—syphilitic or otherwise, accounted for, would we not in such a condition expect those tissues with the least power of resistance or those less stably organised to show the first signs of disturbance ? The muscle-sense, being one of those highly specialised functions and one of a more recent biological development, any neurotic degeneration would, according to one of nature's soundest laws, assail this weak point first. Now, taking it for granted the neuron element is affected by a condition of lowered nutrition, where would we look for its earliest manifestations ? In diseases of the heart and in old age we find the earliest changes in those parts farthest removed from the center of nutrition.

So it is with the changes taking place as a result of lowered nervous vitality. It has been demonstrated by Schafer, Sherrington and Gowers, that the extremities of the neuron element or those parts farthest removed from the neuron-body, *i. e.*, the center of nutrition in the spinal ganglion—are the first to show signs of degeneration or derangement of function. Interference of nutrition in the trophic centers or neuron-bodies, therefore, accounts for the failure of function in the terminals, producing on the one hand peripheral symptoms, such as loss of knee-jerk, loss of sensation, lightning pains and the like, and on the other hand spinal symptoms, such as optic atrophy, ptosis, visceral crises and the like.

Following these functional changes there comes a degeneration of the fibers within the muscle-spindle and the terminal axes-cylinders of the neuraxons and as the disease progresses there is an extension of the degeneration and secondary sclerosis along the path of these nerves. As it has been pointed out before, the path of these nerves, after leaving the spinal roots, enter the cord at Burdach's column and in the lumbar cord cross over to Gall's column. This accounts for the changes found in these portions of the cord in the later stages of the disease. But it must be remembered that the sclerosis is a secondary manifestation and not a primary sclerosis, as was heretofore held.

The primary sclerotic theory had for its justification the post mortem findings of such cases, but these signs of sclerosis were only found in old cases far advanced into the ataxic stage. It was notable that in the early stages the changes found in the cord were conspicuous by their absence. The absence of any pathological signs in the cord in the early stages naturally directed the investigator's attention to other parts of the nervous system. So in Osler's Practice of Medicine of 1892 we find the statement that " perhaps the disease begins with a neuritis of the posterior roots within the vertebral canal. Research was further advanced by Sherrington, 1895, and Batten, 1897, both of whom brought forth ample experimental and anatomical proof that locomotor ataxia begins in a degeneration of the nerve endings of the neuron within the muscle-spindle. Although no absolute proof is forthcoming that the degenerative changes also take place at this time in the upper end of the neuron in the oblongata, yet as the tendency of degenerations is to begin at parts farthest removed from the centers of nutrition, we may safely infer that there is a simultaneous degeneration at both extremities of the neuron.

*Treatment.*—A disease marked by pathological changes, ranging from a mere nutritional disturbance in the first stages to a sclerosis and destruction of nerve elements in the late stages, ought surely to hold out some hopeful therapeutic measures; and more especially as the first stage is not merely transitory and likely to be overlooked. In fact, the first or pre-ataxic stage usually covers a long period of time and gives plenty of warning by a variety of symptoms not easily misinterpreted.

The therapeutic measures are directed towards the reëstablishment of the nutrition of the neuron-element. The four main factors of treatment are diet, hygiene, rest and electricity. The diet should be liberal—meats, fats, milk and fruits, with considerable restriction on the starchy and saccharine foods. Alcohol should be prohibited. If possible, patient should avoid the extremes of heat and cold; should wear warm clothing and the action of the skin, kidneys and bowels carefully looked after. When possible complete rest in bed is advisable, but where not practicable a few hours during the day should be given to sleep. All mental strain and sources of worry should be eliminated.

Drugs, especially those of nutritive value, are useful, such as phosphorus, iron, cod liver oil and strychnia. For lightning pains, both aluminum chloride and methylene blue have been used with good results. Electricity is of the greatest value, if properly employed. The galvanic current alone gives good results. A current of high voltage and low ampherage, administered by means of a foot plate and neck electrode, is the most beneficial. The sittings should last for ten minutes and be repeated every alternate day.

A course of treatment, such as outlined above, should be continued for from four to six weeks and be repeated once or twice yearly. Under this treatment all cases show marked improvement, some more than others, depending on the stage of the disease.

Before closing this brief summary, I wish to invite attention to the recent mode of treatment instituted by Frenkel, of Switzerland, especially adapted to cases advanced into the ataxic stage. The idea of this treatment is to teach the patient, again by a systematic training, the coördination of muscular action that he has lost. The patient is instructed to place the hand or foot on a certain spot and practise at it until he can accomplish such action with exactitude. After one accurate movement is learned, another is tried, and so on. It is claimed for this method that walking and other voluntary acts can be accomplished with precision, even after years of bed-ridden helplessness.

An article of this form of treatment, well worth reading, can be found in the February *Therapeutic Gazette.*

In concluding this brief sketch I wish to lay special stress on the necessity of an early diagnosis. Therefore, in all cases of so-called rheumatism, accompanied by pains, not strictly articular and in cases of disturbed vision, we should keep tabes in mind and a proper investigation will often be the means of warding off one of the most dreaded of neurotic complaints.

550 MAIN STREET.

